# Diagnostic Challenge in Bipolar I Disorder: Idiopathic Parkinson’s Disease vs. Antipsychotic-Induced Parkinsonism vs. Lithium Tremor

**DOI:** 10.1192/j.eurpsy.2026.11790

**Published:** 2026-07-31

**Authors:** S. Adas, E. Jetter, B. Carr

**Affiliations:** University of Florida, Gainesville, United States

## Abstract

**Introduction:**

Tremor in patients with bipolar disorder is frequently attributed to psychotropic medications, especially lithium and antipsychotics, which can obscure recognition of an underlying neurodegenerative disorder. Lithium is classically associated with a fine postural or action tremor, while dopamine D2 receptor blockade by antipsychotics may induce symmetric parkinsonism with resting tremor. By contrast, idiopathic Parkinson’s disease (PD) reflects dopaminergic degeneration and typically presents with asymmetric resting tremor. Diagnostic uncertainty arises when these mechanisms overlap, complicating therapeutic decisions in patients who require both mood stabilization and management of motor symptoms.

**Objectives:**

To describe a case in which lithium tremor, antipsychotic-induced parkinsonism, and idiopathic PD coexisted, illustrating diagnostic pitfalls and therapeutic complexities in the management of bipolar I disorder.

**Methods:**

A middle-aged woman with bipolar I disorder and comorbid restless legs syndrome was maintained on lithium carbonate (450 mg twice daily) and lamotrigine (200 mg twice daily). She developed recurrent lithium toxicity (serum levels >2.0 mmol/L) with tremor and rigidity, prompting lithium discontinuation. Aripiprazole (5-10 mg/day) was initiated, yet parkinsonian features persisted. Neurological evaluation included dopamine transporter (DAT) imaging.

**Results:**

Persistent tremor despite lithium discontinuation, together with ongoing aripiprazole exposure, prior antipsychotic history, and asymmetric reduction of DAT binding in the putamen, supported a diagnosis of idiopathic PD with superimposed drug-induced parkinsonism. Carbidopa-levodopa (25/100 mg three times daily) improved motor function but precipitated hypomanic symptoms. Discontinuation of aripiprazole alleviated rigidity but destabilized mood. Subsequent treatment with quetiapine (titrated to 450 mg nightly), venlafaxine (up to 225 mg/day), and bupropion (150 mg/day) achieved partial mood stabilization, though residual apathy and fatigue remained.

**Conclusions:**

This case demonstrates how overlapping etiologies such as lithium tremor, antipsychotic-induced parkinsonism, and idiopathic PD, can complicate diagnostic clarity in bipolar disorder. Asymmetric DAT findings pointed to a neurodegenerative process, but concurrent medication effects obscured interpretation. Clinicians must balance the need for dopaminergic therapy against the risk of mood destabilization, highlighting the challenge of treating comorbid PD in patients with severe psychiatric illness.

**Disclosure of Interest:**

None Declared

